# Learning-Enhanced Belief Rule Base for Sensor-Based Bearing Health Condition Assessment

**DOI:** 10.3390/s26206447

**Published:** 2026-10-12

**Authors:** Xinrong Li, Yan Li, Shiming Li, Yuhe Wang

**Affiliations:** College of Computer Science and Information Engineering, Harbin Normal University, Harbin 150025, China; 20243007014@stu.hrbnu.edu.cn (X.L.); shimingli@hrbnu.edu.cn (S.L.)

**Keywords:** belief rule base, evidential reasoning, rule learning, fault diagnosis

## Abstract

The Belief Rule Base (BRB) provides explainable reasoning and uncertainty modeling capabilities, offering significant advantages in complex system modeling. However, the scope of its knowledge base is constrained by expert prior knowledge, rendering it incapable of encompassing fault categories that appear in the training data but lack expert-defined rules, thereby preventing post-construction expansion in line with data evolution. To this end, this paper proposes a learning-enhanced BRB model (LE-BRB), achieving a verifiable extension of the rule conclusion space within the BRB framework. First, association rules are introduced to mine classes with missing expert rules and generate candidate rules; secondly, a quality verification module is constructed through Pareto-based filtering, and rule fusion is implemented based on antecedent similarity and conclusion conflict degree; Finally, evidential reasoning is employed to achieve traceable diagnosis. Experiments on the CWRU local bearing fault dataset show that, while preserving the original interpretability of the BRB, LE-BRB completes the missing rolling element fault (Ball) rules in the knowledge base, achieving an average accuracy of 91.02%. Experiments on the MAFAULDA bearing dataset show that, while preserving the original interpretability of the BRB, LE-BRB completes the missing rotor unbalance fault in the knowledge base. This fills the coverage gap of the original BRB for this fault category, improving the diagnostic accuracy from 43.69% to 94.17%. Compared with models such as neural networks and decision trees, it achieves comparable or superior accuracy while exhibiting relatively higher stability.

## 1. Introduction

Rolling bearings constitute a critical component of rotating machinery, and their operational state directly influences industrial safety and production efficiency [1]. Therefore, research on bearing condition monitoring and fault-diagnosis technologies under complex service conditions constitutes a critical foundation for ensuring the long-term stable operation of equipment [2]. However, the fault modes of rolling bearings evolve continuously with service time and operating conditions, and the scarcity of high-quality labeled samples imposes higher demands on the interpretability of diagnostic models, their adaptability to few-shot scenarios, and the autonomous updating capability of the knowledge base [3].

To meet the demand for bearing fault diagnosis, existing research has developed a variety of diagnostic methods. Based on the interpretability of the model architecture, models can be broadly classified into black-box and white-box categories. Black-box models, exemplified by data-driven machine learning and deep learning, possess robust feature-extraction and nonlinear-fitting capabilities [4,5], achieving high diagnostic accuracy in bearing-fault diagnosis. However, the decision-making process lacks an interpretable reasoning structure, and diagnostic conclusions are difficult to trace back. White-box models exemplified by decision trees and fuzzy logic [6] construct transparent decision chains; however, their rule-expressive capacity is often constrained when confronted with high-dimensional continuous features and non-linear coupled faults, rendering it difficult to simultaneously achieve high accuracy and interpretability. In recent years, researchers have introduced enhanced white-box models that incorporate neural networks [7,8,9,10], seeking to improve diagnostic accuracy while maintaining interpretability; however, these approaches still encounter challenges such as high computational complexity and inflexible rule structures. In addition to algorithmic improvements, innovations in sensing hardware also provide new avenues for bearing condition monitoring.

Recently, energy-harvesting-based self-powered sensing has evolved from a mere power-supply solution into an integrated “energy harvesting and self-sensing” hardware paradigm. For example, Qin et al. proposed an ultrasensitive self-powered smart bearing pedestal with fault-locating capability, which uses segmented piezoelectric stacks to harvest energy from rolling-element contact loads and achieves both self-powering and high-sensitivity fault detection [11]. Xiao et al. developed a compact smart bearing with a piezoelectric transducer ring, which enables multi-directional data acquisition and weak defect identification via hybrid feature assessment [12]. Based on triboelectric nanogenerators (TENGs), Li et al. designed a TENG-based smart bearing with self-powered sensing, and proposed a fault diagnosis method by fusing vibration and triboelectric signals, which significantly improves diagnostic accuracy under various noise conditions [13]. Wang et al. proposed a piezoelectric transducer ring-based energy harvesting MEMS (PTR-EH-MEMS) for shaft-bearing monitoring, which converts mechanical vibrations into electrical energy and simultaneously acts as a self-powered sensing element, achieving a maximum DC output of 0.8 V and an RMS power of 43.979 μW [14]. Shang and Xu evaluated the feasibility of using nonlinear piezoelectric energy harvesters for bearing fault diagnosis in a “self-sensing” manner, where the voltage outputs are directly used with deep-learning features to classify bearing fault location and severity [15]. Moreover, Dong et al. developed an AI-enabled rolling triboelectric nanogenerator for bearing wear diagnosis, achieving high-accuracy classification of wear types, degrees, and positions, pointing toward digital twin applications [16]. Collectively, these advances indicate that energy harvesting is transitioning into an integrated sensing-and-diagnosis enabler, providing higher-quality signals for bearing monitoring. However, reliable fault diagnosis still requires an algorithm that can handle multiple uncertainties and maintain transparent, traceable reasoning. The Belief Rule Base (BRB) is exactly such a method.

The Belief Rule Base (BRB) simultaneously ensures diagnostic precision and reasoning transparency, and possesses a formal capacity to handle multiple forms of uncertainty. Its reasoning engine’s evidence reasoning (ER) algorithm is mathematically traceable to the basic probability assignment and orthogonal sum rule of Dempster–Shafer evidence theory [17]; it provides a formal capability to simultaneously handle uncertainty and generate interpretable confidence distributions, and has been validated as effective in multiple equipment-diagnosis scenarios [18,19]. These characteristics render BRB especially suitable for sensor-based fault diagnosis, wherein measurement noise and incomplete observations introduce multiple forms of uncertainty. In terms of knowledge representation, each rule’s antecedent and the confidence distribution of its consequent possess a well-defined semantic interpretation. At the inference decision level, the ER algorithm iteratively synthesizes the confidence distributions of activated rules, preserving the complete mass function at each fusion step, thereby allowing diagnostic conclusions to be traced back to specific rules and their respective contributions.

In recent years, enhancements to BRB have been pursued across dimensions such as sliding-window discretization of vibration signals and dual-frequency feature extraction [20], hierarchical structural design [21], fault-tree analysis [22], attribute reliability [23,24], and interval structures [25], thereby improving the inference accuracy and robustness of BRB within the established framework. However, the aforementioned improvements have not addressed an inherent limitation of the BRB, whose conclusion space is predefined by experts during the modeling phase and cannot autonomously expand as data evolve after the knowledge base is constructed. The extended belief rule base [26] (EBRB) attempts to automatically generate rules from data, but its generation mechanism still requires that all conclusion types be predefined at the modeling stage; if the training data contain fault categories not included in this predefined set, EBRB is unable to recognize them or modify its conclusion space accordingly; the compact belief rule model [27] likewise fails to overcome this structural constraint. In other words, the existing BRB framework lacks a formal method that, while preserving the axioms of evidential reasoning, systematically incorporates sensor patterns corresponding to fault categories that are known from the data taxonomy yet lack expert-defined rules into the set of rule conclusions.

To address the aforementioned challenges, this paper proposes a learning-enhanced belief rule base (LE-BRB) diagnostic model that preserves the complete interpretability of the BRB reasoning process while establishing a traceable selection and resolution mechanism for rule generation and integration. Specifically, its rule addition is supported by explicit validation criteria; candidate rules must undergo Pareto-optimal selection and conflict resolution before being admitted to the rule base. This design enables LE-BRB to autonomously expand its knowledge base while preserving the interpretability of the diagnostic process. First, a data-driven rule generation mechanism based on association rule mining [28] is introduced for fault categories with known labels but missing expert rules. This mechanism automatically extracts candidate rules. Second, a multi-dimensional quality verification module is built through Pareto-based filtering screening and novelty assessment to ensure the reliability and interpretability of the rules. In the final step, the principles and knowledge of the experts are combined to form a set of rules, and the diagnosis of the fault is completed using a method of reasoning based on evidence.

Experiments on the CWRU local bearing fault dataset show that LE-BRB raises the average accuracy from 80.16% for the initial BRB to 91.02%, and achieves an F1-score of 0.65 on the missing Ball class; the EBRB baselines with a closed four-class set and a rejection-based three-class set reach 89.81% and 75.87%, respectively. On the MAFAULDA first dataset, LE-BRB recovers the missing unbalance category and improves the overall accuracy from 43.69% to 94.17%, with an F1-score of 0.99 on the unbalance class. On the MAFAULDA second dataset, it maintains an accuracy of 93.50% and an F1-score of 0.96 on the unbalance class. Across the three datasets, the standard deviation of LE-BRB remains within 0.0013–0.0051, which is among the lowest among the compared models, while its diagnostic conclusions remain traceable to specific rules and their contributions.

## 2. Problem Description

Mechanical equipment fault diagnosis faces multiple challenges, including diverse fault types with complex feature mappings, small-sample problems arising from the high cost of obtaining fault samples, and the requirement for diagnostic results to be interpretable. The Belief Rule Base [17] (BRB), a semi-quantitative information fusion method, was first proposed by Yang et al. Its core rule adopts an ‘IF-THEN’ form, and a belief distribution is introduced in the conclusion part. This structure enables it to simultaneously express fuzziness, randomness, and epistemic uncertainty. Let the input sample be $x=\left(x_{1},\ldots ,x_{M}\right)\in R^{M}$, where *M* is the feature dimension; then the *k*th rule in the BRB can be expressed as:

(1) $$R_{k}:\mathrm{If}\;x_{1}\;\mathrm{is}\;A_{1}^{k}\wedge \ldots \wedge x_{M}\;\mathrm{is}\;A_{M}^{k}\;\mathrm{Then}:\;\left\{\left(D_{1},\beta_{k,1}\right),\ldots ,\left(D_{N},\beta_{k,N}\right)\right\}$$

where *N* is the total number of fault categories; $\beta_{k,n}$ denotes the confidence degree of conclusion $D_{n}$ in the *k*th rule.

However, traditional BRB models face two key issues in practical applications, which restrict their diagnostic performance in scenarios where prior knowledge is incomplete.

Problem 1: Missing conclusions and rules.

Let $R_{\exp}$ be the rule base predefined by experts, let the fault-type set covered by expert knowledge be $D_{\exp}=\left\{D_{1},D_{2},\ldots ,D_{m}\right\}$, and let the set of all fault labels actually appearing in the historical dataset be $D_{\mathrm{lab}}=\left\{D_{1},D_{2},\ldots ,D_{m+n}\right\}$. The set of fault categories for which corresponding rules are missing from the expert knowledge base can then be obtained by the set difference operation:

(2) $$D_{\mathrm{miss}}=D_{\mathrm{lab}}\setminus D_{-}\exp=\left\{D_{m+1},D_{m+2},\ldots ,D_{m+n}\right\}$$

The rule base $R_{\exp}$ of a conventional BRB contains only rules for $D_{\exp}$. For any test sample *x* whose true label satisfies $y\in D_{\mathrm{miss}}$ and $y\notin D_{\exp}$, the model cannot find a rule whose antecedent matches, which results in the following:

(3) $$\hat{y}=\arg\max_{D_{i}\in D_{\exp}}\beta_{i}\left(x\right)\;\mathrm{and}\;\hat{y}\neq y\;\mathrm{or}\;\max_{i}\beta_{i}\left(x\right)\ll 1$$

When $D_{\mathrm{miss}}\neq ⌀$, the classification mapping $f:X\to D_{\exp}$ cannot cover the true label space, resulting in a knowledge coverage gap.

Problem 2: Rule conflicts.

Data-driven algorithms can generate candidate rules for missing faults, but how to integrate these candidate rules into the knowledge base in a reasonable manner remains an open issue. Existing research on BRB fusion predominantly focuses on heuristic adjustments such as rule weights and lacks quantitative conflict metrics and a systematic adaptive resolution mechanism. When the premises of a new rule are highly similar to those of an expert rule but the conclusions conflict, forced insertion will compromise the consistency of the knowledge base [22,29].

To quantitatively characterise conflict phenomena between rules, the premise similarity of two rules $R_{i},R_{j}$ is defined as follows:

(4) $$Sim\left(P_{i},P_{j}\right)=\frac{1}{M}\sum_{l=1}^{M}I\left[A_{l}^{i}=A_{l}^{j}\right]$$

When $Sim\left(P_{i},P_{j}\right)\geq \tau_{sim}$, a conflict exists if the confidence distributions of their conclusions differ considerably. The degree of conflict is as follows:

(5) $$Conflict\left(R_{i},R_{j}\right)=1-\sum_{n=1}^{N}\sqrt{\beta_{i,n}\cdot \beta_{j,n}}$$

If $Conflict(R_{i},R_{j})>\eta$, directly adding the candidate rule would undermine the consistency of the knowledge base. Existing methods lack effective strategies for detecting and resolving such conflicts.

## 3. Methodology

**Figure 1:**
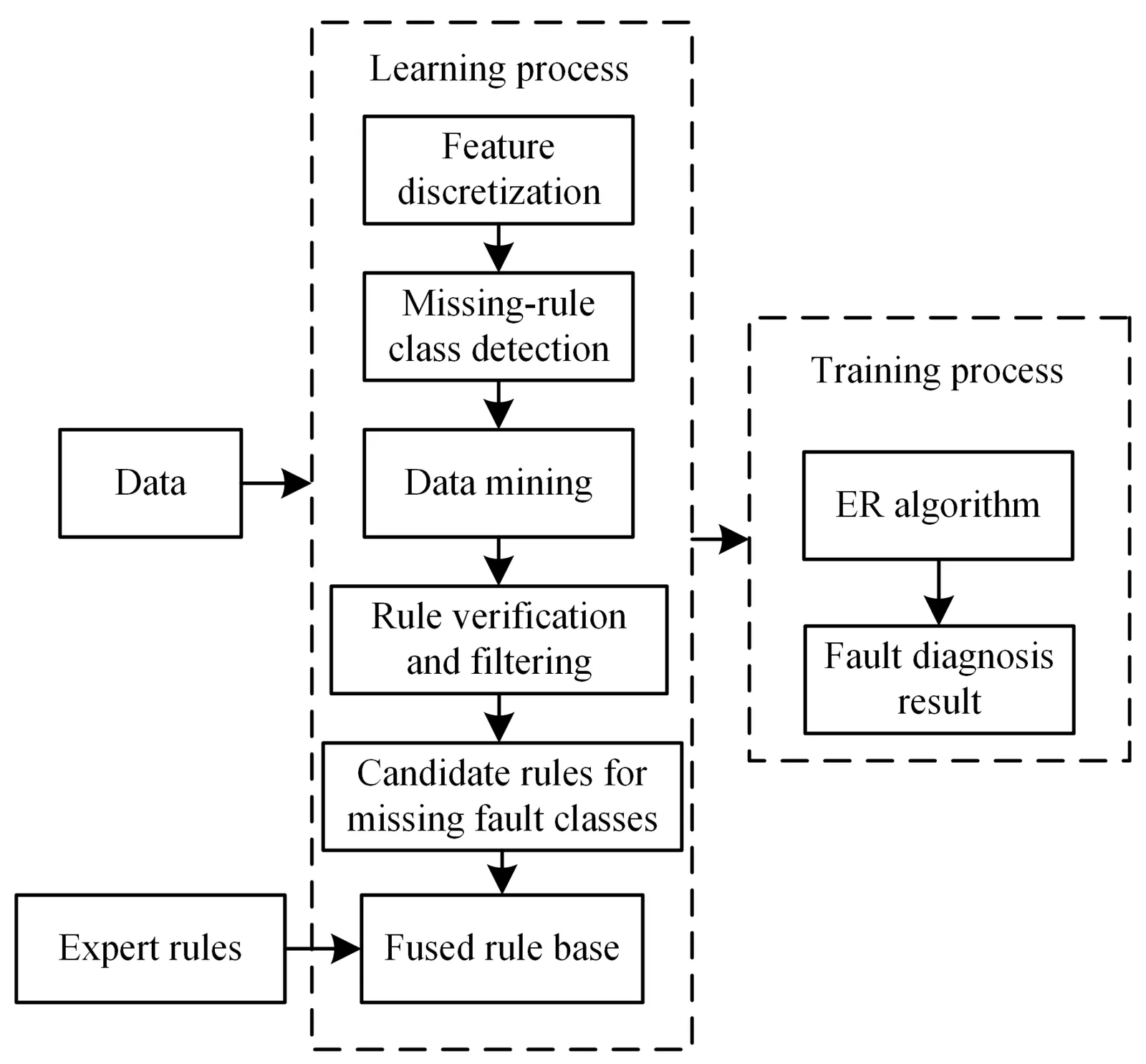
LE-BRB model framework.

The structure of the LE-BRB model proposed in this paper consists of two parts, as shown in Figure 1.

During the learning process, key features are extracted from the information and discretized into linguistic values according to prior knowledge; association rule algorithms are then used to learn new rules from historical data, and rule verification and quality screening ensure the reliability of the learned rules; finally, a fusion algorithm is employed to integrate the learning rules with the expert rules, forming an enhanced belief rule base. During the inference process, evidential reasoning is used to combine the matched results and obtain the final fault diagnosis result.

### 3.1. Learning Process Based on Association Rule Mining

**Figure 2:**
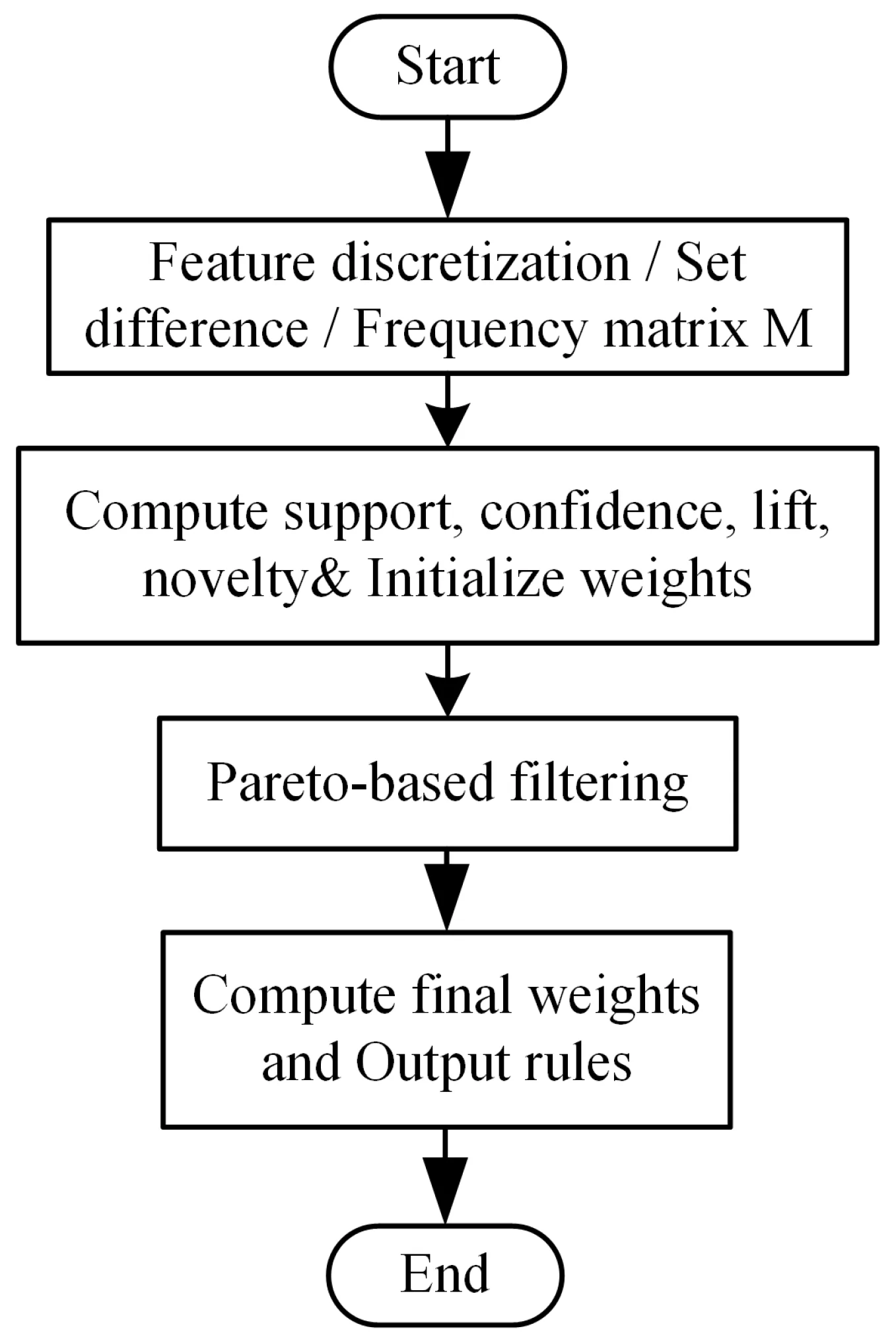
Data-driven rule mining process.

To automatically supplement the lack of expert-defined fault knowledge from data, LE-BRB adopts the idea of association rule mining to generate data-driven candidate rules; the process of which is shown in Figure 2.

As described in Section 1, obtain the set of missing categories $D_{\mathrm{miss}}$ of the knowledge base.

After determining the missing fault categories in the knowledge base, the model extracts dual-feature data from the sample set for each category of knowledge-base-missing fault to perform pattern mining. Let the sample set of the target fault type $D_{m}$ be $S\;=\;\{x^{\left(1\right)},x^{\left(2\right)},\ldots ,x^{\left(n\right)}\}$, where each sample $x^{\left(i\right)}=\left(x_{1}^{\left(i\right)},x_{2}^{\left(i\right)}\right)\in R^{2}$ contains two feature values.

Based on an expert semantic partition, discretization mapping functions $\phi_{1}$ and $\phi_{2}$ are defined to map continuous feature values to a finite semantic set:

(6) $$\phi_{1}:R\to V_{1}=\left\{v_{1,1},v_{1,2},\ldots ,v_{1,m}\right\},\;\phi_{2}:R\to V_{2}=\left\{v_{2,1},v_{2,2},\ldots ,v_{2,k}\right\}$$

Here $F_{1}$ and $F_{2}$ denote the names of the two input features, that is, the physical quantities corresponding to $x_{1}$ and $x_{2}$; $V_{1}$ and $V_{2}$ are the sets of semantic values of features $F_{1}$ and $F_{2}$, whose cardinalities are $m\;=\;|V_{1}|$ and $k\;=\;|V_{2}|$, respectively.

Construct a frequency statistics matrix $M\in N^{m\times k}$, whose elements are as follows:

(7) $$m_{ab}=\sum_{i=1}^{n}I\left[\phi_{1}\left(X_{1}^{\left(i\right)}\right)=v_{1,a}\wedge \phi_{2}\left(X_{2}^{\left(i\right)}\right)=v_{2,b}\right]$$

Here I[·] is the indicator function.

Based on the frequency matrix, the support matrix $S\in R^{m\times k}$ is further calculated for the pattern $P_{ab}=\left\{\left(F_{1},v_{1,a}\right),\left(F_{2},v_{2,b}\right)\right\}\to D_{\mathrm{miss}}$ formed by each group of semantic combinations $\left(v_{1,a},v_{2,b}\right)$:

(8) $$s_{ab}=\frac{m_{ab}}{n}$$

To convert frequent patterns into standard confidence rules, this paper employs a frequency statistics method based on Laplace smoothing for the quantification of confidence distributions. Let the confidence of the *k*th fault category among the samples satisfying the current pattern premise be $\beta_{k}^{\left(P\right)}$; its calculation is then given by the following:

(9) $$\beta_{k}\left(P\right)=\frac{\sum I[y^{\left(i\right)}=D_{k}]+\lambda }{|S_{P}|\;+\lambda \cdot N}$$

Here, $S_{P}$ is the set of samples matching the current premise, $y^{\left(i\right)}$ is the true label of a sample, and λ is the smoothing coefficient.

After confidence quantification is completed, the initial weight of each newly generated data rule is positively correlated with its coverage. The initial weight of a data rule is calculated as follows:

(10) $$\theta_{\mathrm{data}}=\min\left(0.9,\;0.5+0.4\times \frac{|S_{P}|}{N_{D_{m}}}\right)$$

where $N_{D_{m}}$ is the total number of samples of the target rule-missing category in the training set.

To select rules that perform well across multiple quality indicators from the candidate rule set, this paper adopts Pareto-based filtering.

To measure the knowledge gain of the data-driven rules relative to existing expert knowledge, a novelty metric is defined. Let the premise semantic vector of rule *R* be $a=(a_{1},a_{2})$, and let the premise semantic vector of rule $R_{\exp}$ in the expert rule base $R_{\exp}$ be $a_{\exp}$. The simple matching coefficient is used to calculate the premise similarity:

(11) $$MatchSim\left(a,a_{\exp}\right)=\frac{\left|\{i|a_{i}=a_{exp,i}\}\right|}{M}$$

where M=2 is the number of features.

The novelty of rule *R* is as follows:

(12) $$Q_{\mathrm{novelty}}\left(R\right)=1-\max_{a_{\exp}\in R_{\exp}}MatchSim\left(a,a_{\exp}\right)$$

$Q_{\mathrm{novelty}}\in \left[0,1\right]$, where a larger value indicates a greater deviation from existing expert knowledge.

After novelty scoring, the remaining candidate rules proceed to multi-criteria screening.

For each candidate rule $R_{cand}$, its three core quality indicators are calculated: support $s=\frac{|S_{P}|}{\left|S\right|}$, confidence $c=\beta_{D_{m}}\left(P\right)$, and lift $l=\frac{c}{p_{D_{m}}}$.

To automatically select high-quality rules under multiple potentially conflicting metrics, the Pareto-based filtering concept is introduced. Rule $R_{i}$ dominates rule $R_{j}$ (denoted $R_{i}≺R_{j}$) if and only if $s_{i}\geq s_{j}$, $c_{i}\geq c_{j}$, and $l_{i}\geq l_{j}$ are satisfied, and at least one of the inequalities holds strictly.

The Pareto-optimal front consists of all rules that are not dominated by any other rule, that is as follows:

(13) $$P*=\left\{R\in P|∄R^{\prime }\in P,\;R^{\prime }≺R\right\}$$

Rules on the Pareto front are non-dominated with respect to all three metrics; that is, there exists no other rule that is no worse on all metrics and strictly better on at least one metric. These rules are directly admitted to the passing set. Therefore, the verified rule set is as follows:

(14) $$R_{\mathrm{valid}}=P*$$

Support quality: $Q_{s}=\min\left(1,\frac{|S_{P}|}{50}\right)$;

Belief concentration: the entropy of the rule conclusion distribution is $H=-\sum_{k=1}^{N}\beta_{k}\log_{2}\beta_{k}$, and thus $Q_{c}=1-\frac{H}{\log_{2}N}$;

The comprehensive quality score of a rule is calculated by integrating its support quality, belief concentration and novelty:

(15) $$Q=w_{s}Q_{s}+w_{c}Q_{c}+w_{n}Q_{\mathrm{novelty}}$$

where $Q_{s}$ is the support quality, $Q_{c}$ is the belief concentration, and $Q_{\mathrm{novelty}}$ is the novelty. The weight vector is taken as $\left(w_{s},w_{c},w_{n}\right)=\left(0.35,0.35,0.30\right)$. Therefore, novelty does not change the composition of the Pareto front and is only used for weight calibration of rules that have passed the screening. The final rule weight mapping is expressed as follows: θ=0.35+0.50·Q.

After data rule verification is completed, the rule set $R_{\mathrm{valid}}$ generated through data-driven methods must be fused with the initial expert rule base $R_{\exp}$, and the core of the fusion process lies in conflict detection and resolution between rules. To quantitatively describe the degree of similarity between rule antecedents, an antecedent similarity index is defined. For a rule antecedent with *M* attributes, the similarity is calculated as follows:

(16) $$Sim\left(P_{i},P_{j}\right)=\frac{1}{M}\sum_{l=1}^{M}I\left[A_{I}^{i}=A_{l}^{j}\right]$$

Given a threshold τ∈(0,1], if $Sim\left(P_{i},P_{j}\right)\geq \tau_{sim}$, the premises are regarded as highly similar. The conclusion conflict degree is further defined as follows:

(17) $$Conflict\left(R_{i},R_{j}\right)=1-\sum_{k=1}^{N}\sqrt{\beta_{i,k}\cdot \beta_{j,k}}$$

where η∈(0,1) is the conflict threshold, determined by the number of fault categories *N* and semantic discretization.

The rationale for conflict resolution is as follows: data-driven rules may be biased due to noise or limited samples, whereas expert rules encode domain prior knowledge and are generally more reliable. Therefore, three cases are considered:

If $Sim(P_{i},P_{j})=1$ and $Conflict(R_{i},R_{j})>\eta$, the data rule and the expert rule share the same premise but lead to different conclusions. The conflict arises from inconsistent belief distributions. In this case, the conservative strategy prioritizes expert knowledge to preserve the consistency of the knowledge base. The expert rule $R_{j}$ is retained and the weight of the data rule is reduced:

(18) $$\theta_{i}\leftarrow \alpha \cdot \theta_{i},\;\alpha \in \left(0,1\right)$$

If $\tau_{sim}<Sim\left(P_{i},P_{j}\right)<1$ and $Conflict(R_{i},R_{j})>\eta$, the data rule partially overlaps with the expert rule and also introduces new information that deviates from the expert knowledge. In this case, fusion is preferred over simple retention or rejection, so that the knowledge base can adapt to new data patterns while maintaining consistency. A new rule $R_{fused}$ is generated by fusion:

(19) $$\beta_{\mathrm{fused},n}=\frac{\theta_{i}\cdot \beta_{i,n}+\theta_{j}\cdot \beta_{j,n}}{\theta_{i}+\theta_{j}},\;n=1,\ldots ,N$$

(20) $$\theta_{fused}=\frac{\theta_{i}+\theta_{j}}{2}$$

Otherwise, when the premise similarity is below $\tau_{sim}$ or the conflict degree is below the threshold, the data rule and the expert rule are sufficiently different, and both are retained without modification. This allows the knowledge base to expand its coverage without disturbing existing expert knowledge.

To systematically describe the entire process of candidate rule generation, selection and fusion, this section presents the complete algorithm for LE-BRB rule learning, as detailed in Algorithm 1. The algorithm takes the training sample set and the expert rule base as inputs, sequentially performs five steps: identification of faults missing from the knowledge base, candidate rule generation, Pareto-based filtering, rule weight mapping and conflict resolution, and finally outputs the fused enhanced rule base.

Lines 1–17 correspond to the recognition of knowledge base missing faults and candidate rule generation in Equations (2)–(10); lines 18–26 correspond to the Pareto-based filtering and rule weight mapping in Equations (11)–(15); line 27 corresponds to the conflict detection and fusion resolution in Equations (16)–(20).

### 3.2. Diagnostic Decision Based on Evidential Reasoning

For a new input sample $X_{\mathrm{new}}={\left[x_{1},x_{2}\right]}^{T}$, LE-BRB employs the Evidential Reasoning (ER) algorithm based on the enhanced rule base $R_{\mathrm{fused}}$ to perform diagnostic decision-making, a process that can transparently handle the uncertainties in rule matching.

First, the continuous features of $X_{\mathrm{new}}$ are converted into a semantic vector $x^{\left(i\right)}=\left(x_{1}^{\left(i\right)},x_{2}^{\left(i\right)}\right)$ via an expert discretization function. Then, all rules in $R_{\mathrm{fused}}$ whose antecedents exactly match $x^{\left(i\right)}$ are identified to form the activated rule set $R_{act}=\left\{R_{1},R_{2},\ldots ,R_{l}\right\}$.
**Algorithm 1** Data-Driven Candidate Rule Generation and Fusion Algorithm**Require:** Training sample set $S=\{\left(x_{1},y_{1}\right),\left(x_{2},y_{2}\right),\ldots ,\left(x_{n},y_{n}\right)\}$, expert rule base $R_{\exp}$, minimum support threshold $\theta_{\min}$, similarity threshold τ, conflict threshold η, smoothing coefficient λ**Ensure:** Enhanced rule base $R_{\mathrm{fused}}$   1: $R_{\mathrm{fused}}\leftarrow R_{\exp},\;R_{\mathrm{cand}}\leftarrow ⌀$   2: $D_{\mathrm{miss}}\leftarrow D_{\mathrm{lab}}\setminus D_{\exp}$   3: **for** each $D_{u}\in D_{\mathrm{miss}}$ **do**   4:       $S_{u}\leftarrow \left\{x^{\left(i\right)}|y^{\left(i\right)}=D_{u}\right\}$   5:       **for** each $x^{\left(i\right)}\in S_{u}$ **do**   6:     $\left(v_{1,a},v_{2,b}\right)\leftarrow \left(\phi_{1}\left(x_{1}^{\left(i\right)}\right),\phi_{2}\left(x_{2}^{\left(i\right)}\right)\right)$   7:              $C_{ab}\leftarrow C_{ab}+1$   8:       **end for**   9:       **for** each $\left(v_{1,a},v_{2,b}\right)$ with $C_{ab}>0$ **do** 10:              $s_{ab}\leftarrow C_{ab}/\left|S_{u}\right|$ 11:              $\beta \left(P\right)\leftarrow \left(\sum I[y^{\left(i\right)}=D_{k}]+\lambda \right)/\left(|S_{P}|+\lambda \cdot N\right)$ 12:              $l_{ab}\leftarrow \beta \left(P\right)/p\left(D_{u}\right)$ 13:              **if** $s_{ab}\geq \theta_{\min}$ **then** 14:                   $R_{\mathrm{cand}}\leftarrow R_{\mathrm{cand}}\cup \left\{R:\left(v_{1,a},v_{2,b}\right)\to D_{u},s_{ab},\beta \left(P\right),l_{ab}\right\}$ 15:              **end if** 16:       **end for** 17: **end for** 18: $R_{\mathrm{valid}}\leftarrow \mathrm{ParetoFilter}\left(R_{\mathrm{cand}},R_{\exp}\right)$ 19: **for** each $R\in R_{\mathrm{valid}}$ **do** 20:       $Q_{s}\leftarrow \min(1,|S_{P}\left|/50\right)$ 21:       $H\left(R\right)\leftarrow -\sum_{k=1}^{N}\beta_{k}\cdot \log_{2}\left(\beta_{k}\right)$ 22:       $Q_{c}\leftarrow 1-H\left(R\right)/\log_{2}\left(N\right)$ 23:       $Q_{\mathrm{novelty}}\leftarrow 1-\max_{R_{\exp}\in R_{\exp}}\mathrm{MatchSim}\left(a,a_{\exp}\right)$ 24:       $Q\leftarrow 0.35\cdot Q_{s}+0.35\cdot Q_{c}+0.30\cdot Q_{\mathrm{novelty}}$ 25:       $\theta_{R}\leftarrow 0.35+0.50\cdot Q$ 26: **end for** 27: $R_{\mathrm{fused}}\leftarrow \mathrm{ConflictResolve}\left(R_{\exp},R_{\mathrm{valid}},\tau ,\eta \right)$ 28: **return**
$R_{\mathrm{fused}}$

It should be noted that the conclusion space of the expert rule base is $\Theta_{\exp}=\left\{D_{1},D_{2},\ldots ,D_{m}\right\}$, while the data-driven learned rules introduce the missing category $D_{\mathrm{miss}}$, forming the extended conclusion space $\Theta_{\mathrm{new}}=\Theta_{\exp}\cup D_{\mathrm{miss}}$. Since expert rules and learned rules are defined on different frames of discernment, direct ER combination is not valid. To address this, the belief distribution of expert rules on $\Theta_{\exp}$ is mapped to $\Theta_{\mathrm{new}}$ via vacuous extension:

(21) $${\tilde{\beta }}_{j,n}=\left\{\begin{array}{cc} \beta_{j,n}, & n\in \Theta_{\exp},\\ 0, & n\in D_{\mathrm{miss}}, \end{array}\right.\;\sum_{n\in \Theta_{\mathrm{new}}}{\tilde{\beta }}_{j,n}=1.$$

This extension satisfies the refinement condition: the belief distribution on the original frame $\Theta_{\exp}$ remains unchanged, and the belief on new categories is provided solely by the learned rules. Therefore, all activated rules are combined under the unified frame $\Theta_{\mathrm{new}}$ in the subsequent ER process, and the unassigned mass is recalculated on $\Theta_{\mathrm{new}}$.

The contribution of each activation rule $R_{i}$ to the final decision is governed by its rule weight $\theta_{i}$, which is then normalized to yield the activation weight $w_{i}$:

(22) $$w_{i}=\frac{\theta_{i}}{\sum_{j=1}^{L}\theta_{j}+\delta }$$

where δ is a very small positive number used to avoid division by zero.

After obtaining the activation rules and their normalized weights, the belief distributions of multiple rules are iteratively combined using the evidential reasoning algorithm. First, the initial basic mass function is generated by the first rule, including the mass allocated to each fault category and the mass allocated to the frame of discernment:

(23) $$m_{i,n}=w_{i}\cdot \beta_{i,n},\;n=1,\ldots ,N$$

(24) $$m_{i,\Theta }=1-\sum_{n=1}^{N}m_{i,n}=1-w_{i}$$

In the subsequent iterative synthesis process, the degree of conflict between items of evidence is quantified by a conflict coefficient:

(25) $$K_{i}={\left[1-\sum_{p=1}^{N}\sum_{\begin{array}{c} q=1\\ q\neq p \end{array}}^{N}m_{I(i-1),p}\cdot m_{i,q}\right]}^{-1}$$

The mass function is also normalized and updated:

(26) $$m_{I(i),n}=K_{i}\cdot \left[m_{I(i-1),n}\cdot m_{i,n}+m_{I(i-1),n}\cdot m_{i,\Theta }+m_{I(i-1),\Theta }\cdot m_{i,n}\right]$$

(27) $$m_{I(i),\Theta }=K_{i}\cdot \left[m_{I(i-1),\Theta }\cdot m_{i,\Theta }\right]$$

Information from all activated rules is gradually fused. Once all rules have been fused, the synthesized joint mass function is converted into the final confidence for each fault category and the global uncertainty:

(28) $$\beta_{n}=\frac{m_{I(L),n}}{1-m_{I(L),\Theta }},\;n=1,\ldots ,N$$

(29) $$\beta_{\Theta }=\frac{m_{I(L),\Theta }}{1-m_{I(L),\Theta }}$$

Based on the synthesized confidence distribution $\left\{\beta_{1},\ldots ,\beta_{M},\beta_{\Theta }\right\}$, the fault category is predicted using the maximum confidence principle as follows:

(30) $$\hat{y}=\arg\max_{n\in \{1,\ldots ,N\}}\beta_{n}$$

The reasoning process of LE-BRB preserves the full interpretability of BRB. It can be seen from Equations (21)–(29) that the generation of the diagnostic conclusion comprises three transparent steps. First, Equation (21) calculates the normalized weight of each activated rule, clarifying the quantitative contribution of each rule to the final decision. Second, the evidential reasoning algorithm in Equations (22)–(26) iteratively combines belief distributions, where each iteration retains the mass function of each fault category and the global uncertainty, and the intermediate results are auditable. Finally, Equations (27)–(29) output the final belief degree of each fault category and the prediction result. The complete reasoning chain of the diagnostic conclusion can be traced, covering all steps from input features, activation rules, and evidence aggregation to the final decision, with full transparency throughout.

## 4. Experimental Study and Results Analysis

To verify the effectiveness of the proposed learning-enhanced belief rule base (LE-BRB) model, this section conducts a case study using two publicly available mechanical fault datasets. This case demonstrates the capability of LE-BRB in discovering and integrating missing fault categories in the knowledge base, and provides a comparative analysis with traditional models.

### 4.1. Experimental Dataset

To verify the diagnostic performance and generalization capability of LE-BRB on both local bearing faults and system-level faults, three publicly available datasets are used in this study: the CWRU local bearing fault dataset [30] as the main experiment, and the MAFAULDA dataset [31] divided into two sub-datasets according to different load conditions as generalization validation. The class distribution, sample size, and training/testing split of the three datasets are shown in Table 1.

The CWRU dataset is constructed based on the rolling bearing dataset publicly available from the Case Western Reserve University (CWRU) Bearing Data Center. The data were collected from a CWRU rolling bearing fault simulation test rig, which consists of a 1.5 kW (2 hp) Reliance induction motor, a torque sensor/encoder, a power meter, and a dynamometer. Single-point defects were manufactured on the bearings by electro-discharge machining, and accelerometers were mounted on the housing above the drive-end and fan-end bearing housings to collect vibration acceleration signals under multiple operating conditions. The dataset covers four major states: normal, inner race fault, outer race fault, and rolling element fault. The test object is a drive-end deep groove ball bearing, model SKF 6205. Vibration signals were sampled at 12 kHz (with an additional 48 kHz for the drive end).In this study, the drive-end accelerometer signals under four load conditions of 0, 1, 2, and 3 hp were used, and each load condition corresponds to one data file of approximately 20 s (240,000 points). Each file was segmented into frames of 4096 points with a step of 2048 points (i.e., 50% overlap), yielding approximately 116 samples per file. The four files together produce approximately 464 samples, which is consistent with the reported 471 samples for the normal class. The spectral centroid and kurtosis were computed for each frame to obtain a single feature vector per frame. The CWRU dataset contains four classes: Normal, Inner, Outer, and Ball, among which Ball is the missing class in the expert knowledge base. The training and testing sets are split at a ratio of 3:7, and 10-round repeated random subsampling validation is adopted. Due to the limited number of original samples, 5% Gaussian noise is added to all fault classes in the dataset for data augmentation, while the expert rules remain unchanged.

The MAFAULDA dataset was collected using a machinery fault simulator. Six accelerometers, one tachometer, and one microphone were mounted on the bearing housing to acquire vibration, rotational speed, and acoustic signals under multiple operating conditions, covering ten condition types in total, including normal, unbalance, and vertical misalignment. The test object is a bearing of type SKF 6205, with a sampling frequency of 50 kHz, and each operating condition corresponds to one 5 s data file (250,000 points). In this study, the signals from the six accelerometers were used, and each 5 s file was segmented into frames of 0.1 s (5000 points) with 10% overlap, yielding approximately 55 samples per file. The MAFAULDA first dataset serves as the validation set and contains three classes: normal, unbalance, and misalignment, where unbalance is the missing class; it retains the normal condition, two load conditions of vertical misalignment, and three load conditions of unbalance, with approximately 207 files and 11,368 samples, and class proportions of 50.4%, 33.2%, and 16.4%, respectively. The MAFAULDA second dataset serves as the generalization set and also contains three classes, including all normal samples and samples from two different load conditions for each of the two states, with approximately 217 files and 11,956 samples, and class proportions of 41.4%, 38.5%, and 20.1%, respectively. Both datasets are split into training and testing sets at a ratio of 3:7, and 10-round repeated random subsampling validation is employed. The semantic discretization partitions for the first and second MAFAULDA datasets are reset separately according to their own feature distributions, while the rule structure of LE-BRB remains unchanged, so that the generalization performance is evaluated under different operating conditions without altering the model structure.

The Belief Rule Base (BRB) is highly prone to combinatorial explosion when the number of antecedent attributes increases. If there are *T* antecedent attributes and the *i*-th attribute has $J_{i}$ candidate values, the total number of rules is $\prod_{i=1}^{T}J_{i}$. For example, with five antecedent attributes and three candidate values for each attribute, the number of rules reaches $3^{5}=243$; as the feature dimension increases, the rule base size grows exponentially. The most direct and effective way to alleviate this problem is to reduce the number of antecedent attributes, i.e., to select the key performance parameters that are truly critical for the diagnostic task. Therefore, this study selects spectral centroid and kurtosis as diagnostic features. The former characterizes the global distribution of vibration energy in the frequency domain, whilst the latter reflects the impulse characteristics of the time-domain waveform. The two are physically meaningful and complementary, and have been widely adopted in the field of rotating machinery fault diagnosis. Because the numerical boundary between the two is clear, it is convenient for experts to set the semantic grade thresholds of the antecedent attributes of BRB rules on the basis of physical common sense.

Let the original time-domain signal be x(t), its Fourier transform be X(f), and the power spectral density be $P\left(f\right)={\left|X\left(f\right)\right|}^{2}$; the spectral centroid is then defined as follows:

(31) $$F_{centroid}=\frac{\sum_{k=1}^{K}f_{k}\cdot P\left(f_{k}\right)}{\sum_{k=1}^{K}P\left(f_{k}\right)}$$

Here *K* is the number of frequency points in the positive-frequency part. It characterizes the concentrated position of the spectral energy of the vibration signal and reflects the energy distribution characteristics of the system in the frequency domain, serving as a first-order global frequency-domain statistic.

Kurtosis is defined as the ratio of the fourth-order central moment of a signal to the square of its variance:

(32) $$F_{kurtosis}=\frac{\frac{1}{T}\sum_{t=1}^{T}{\left(x_{t}-\mu \right)}^{4}}{{\left(\frac{1}{T}\sum_{t=1}^{T}{\left(x_{t}-\mu \right)}^{2}\right)}^{2}}$$

where μ is the signal mean. Kurtosis is a measure of the sharpness of a vibration signal waveform and is sensitive to impulsive components.

It should be emphasized that the inferential capability of the LE-BRB arises from its rule structure and evidential reasoning mechanism, rather than from the discriminative power of specific features. The criterion for feature selection is that the features possess clear physical meaning and facilitate semantic modeling, rather than maximizing inter-class separability.

Traditional BRB adopts deterministic single-value referential points given by experts based on prior knowledge or historical information. This hard discretization is the basic form of BRB knowledge representation. Since the numerical boundaries of spectral centroid and kurtosis are clear, experts can reasonably set semantic grade thresholds based on physical common sense, thereby directly incorporating domain knowledge into the rule antecedents and endowing the rules with clear physical meaning and interpretability. At the same time, deterministic referential points help control the number of semantic grades, avoid excessive expansion of the rule base size, and facilitate subsequent rule inspection and traceability of diagnostic conclusions. Therefore, hard discretization not only conforms to the modeling paradigm of BRB, but is also sufficient to characterize the faults studied in this paper.

Since their numerical boundaries are clear, it is convenient for experts to set the semantic grade thresholds of the antecedent attributes of BRB rules based on physical common sense. The semantic discretization mapping of CWRU is shown in Table 2; the semantic partitions of the MAFAULDA first and second datasets are set separately due to different operating conditions.

### 4.2. CWRU Local Bearing Fault Experiment

#### 4.2.1. Rule-Level Statistics and Mined Rules

The rule learning and validation process of LE-BRB on CWRU is as follows: 5 candidate patterns are discovered from the Ball-class training samples; 3 rules pass the Pareto front, and 3 are finally accepted, with 2 eliminated. During the generation stage, inconsistency between rule conclusions and premises is detected, and weight adjustments are performed on the 3 rules; the number of conflict detections in the fusion stage is 0, the number of fusions is 0, and the expert rules remain unchanged. The expert rule weight is fixed at 0.9, and the learned rule weights are calibrated to 0.509–0.597. The detailed rule-level statistics are shown in Table 3. Examples of the 3 learned Ball rules are shown in Table 4.

#### 4.2.2. Ablation Study and Comparison with EBRB on the CWRU Dataset

The CWRU dataset contains four classes: Normal, Inner, Outer, and Ball, where Ball is the missing class. The training set has 353 samples (30%), and the testing set has 824 samples (70%); 5% Gaussian noise is added to all fault samples in the dataset, while the expert rules remain unchanged; 10-round repeated random subsampling validation is adopted, where the training and testing sets are re-divided in each round, and the class proportions of the testing set remain unchanged.

To verify the capability of autonomous rule expansion, the initial BRB and two EBRB baselines are set up. In the initial BRB, the expert rules only cover Normal, Inner, and Outer, and the Ball class is missing. One EBRB uses a four-class closed set, and the other EBRB pre-defines only Normal, Inner, and Outer as a three-class open set with rejection.

**Figure 3:**
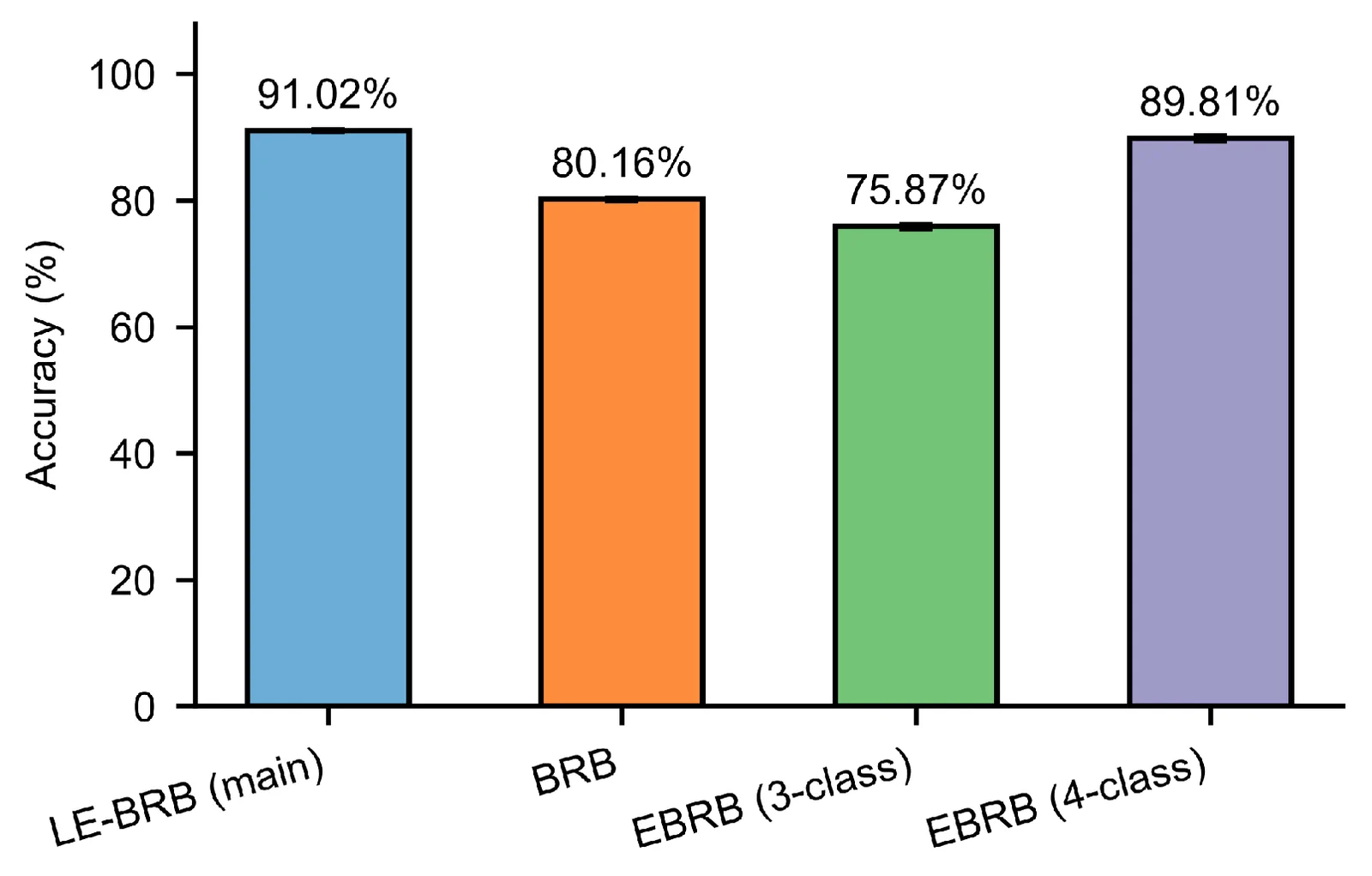
Comparison of LE-BRB and EBRB on CWRU.

As shown in Figure 3, Table 5 and Table 6, the 10-round cross-validation average accuracy of LE-BRB on CWRU is 91.02%, with a coefficient of variation of 0.0056 and a standard error of the mean of 0.0016. Since the initial BRB cannot recognize the missing class Ball, both the F1-score and recall of the Ball class are 0, and all Ball samples are misclassified as Normal, resulting in an overall accuracy of 80.16%. LE-BRB automatically supplements three Ball-related rules through data mining, improving the overall accuracy to 91.02%, and the Ball class F1-score reaches 0.65, indicating that the supplement of missing-class rules is effective. The EBRB with a four-class closed set has pre-defined the Ball class during the modeling stage, and can recognize Ball, with a Ball F1-score of 0.72 and an overall accuracy of 89.81%. For the EBRB with a three-class open set and rejection, since the conclusion space does not include Ball, the Ball rejection rate is 0%, indicating that when the missing class is not pre-defined in the conclusion space, EBRB cannot autonomously supplement rules for this class. In contrast, LE-BRB, without pre-defining expert rules for Ball, can still supplement rules through data mining, verifying the effectiveness of its autonomous rule expansion.

#### 4.2.3. Experimental Comparison on the CWRU Dataset

To verify the diagnostic accuracy and generalization performance of LE-BRB under the premise of interpretability, this section selects six mainstream methods for horizontal comparison, including black-box models represented by purely data-driven approaches: Support Vector Machine (SVM), Multi-Layer Perceptron (MLP) and Random Forest (RF), as well as white-box models driven by rules or cases: Decision Tree (DT), Logistic Regression (LR) and Case-Based Reasoning (CBR). Among them, the decision rules of DT and LR can be explicitly parsed, and CBR performs transparent reasoning based on memory, making them comparable to LE-BRB at the level of knowledge representation. Through multi-dimensional metric comparison, this section aims to comprehensively evaluate the integrated advantages of LE-BRB in terms of accuracy, fine-grained recognition capability, and output stability.

**Figure 4:**
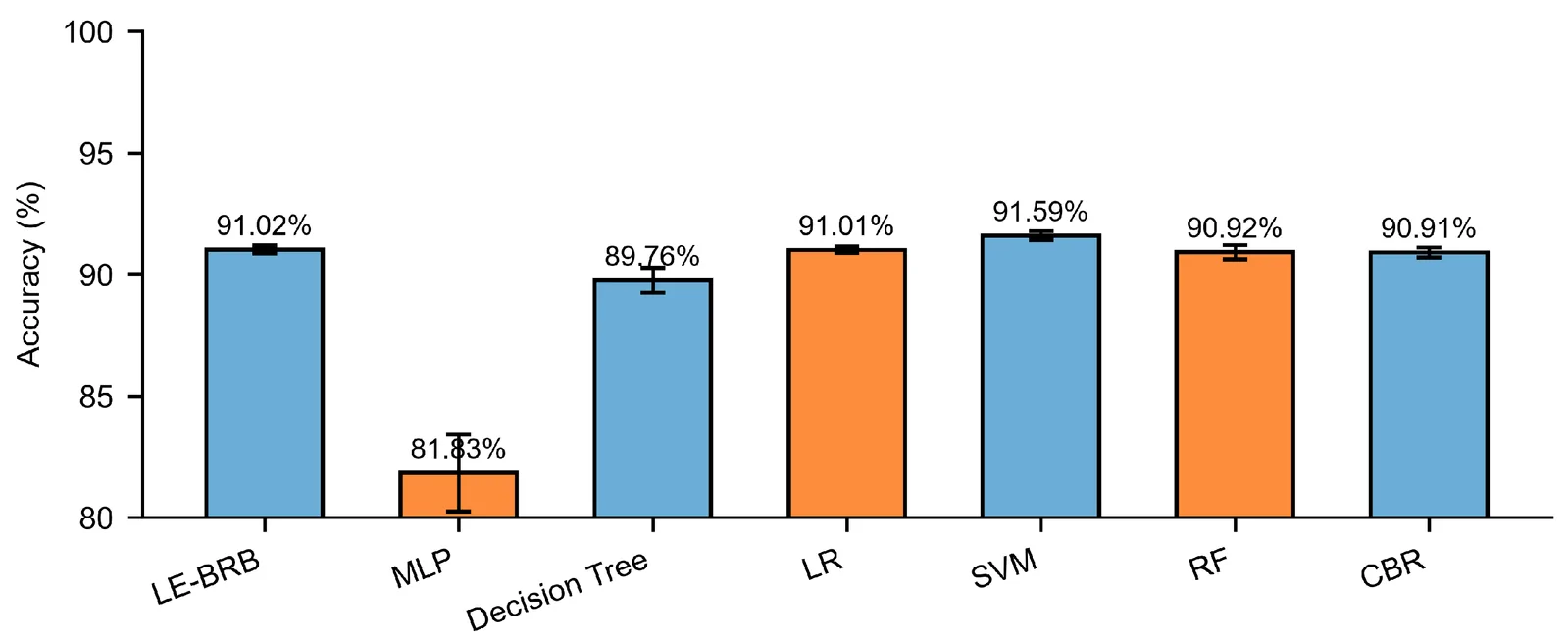
Accuracy comparison on CWRU.

**Figure 5:**
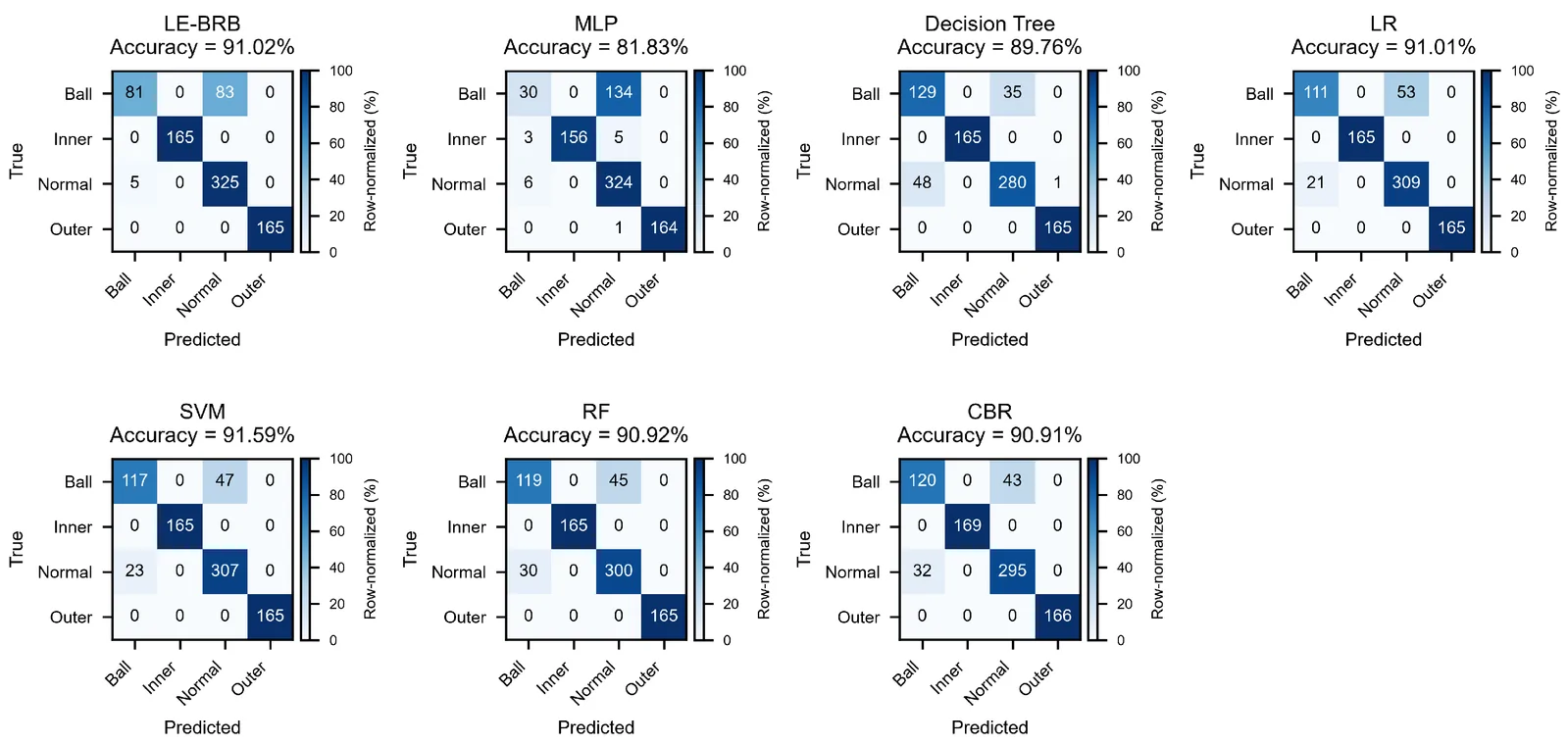
Confusion matrices of different models on CWRU.

As shown in Figure 4, Table 7 and Table 8, the average accuracy of LE-BRB is 91.02%, which is comparable to SVM at 91.59%, LR at 91.01%, and RF at 90.92%, and outperforms MLP at 81.83%, DT at 89.76%, and CBR at 90.91%. In terms of per-class metrics, LE-BRB achieves an F1-score of 0.65 on the Ball class (the missing class), which is slightly lower than RF at 0.76, SVM at 0.77, DT at 0.76, LR at 0.75, and CBR at 0.76, but significantly better than MLP at 0.20. The confusion matrices in Figure 5 further illustrate the misclassification patterns. LE-BRB misclassifies 83 Ball samples as Normal, while RF, SVM, DT, LR, and CBR misclassify 45, 47, 35, 53, and 43 Ball samples as Normal, respectively. Although LE-BRB has a higher number of Ball-to-Normal misclassifications, its overall accuracy remains competitive. For the Inner, Normal, and Outer classes, LE-BRB achieves high F1-scores of 1.00, 0.89, and 0.98, respectively, demonstrating robust performance on known classes.

The standard deviation (SD) and coefficient of variation (CV) of the results from 10 repeated experiments are used as quantitative evaluation metrics for model stability, as shown in Table 9. LE-BRB achieves an SD of 0.0051 and a CV of 0.0060, which are among the lowest values. Although LR and SVM have slightly lower SD and CV, their accuracy is comparable to or slightly higher than LE-BRB. However, LE-BRB maintains a balance between accuracy and stability. Compared with RF, which has a slightly higher accuracy, LE-BRB’s SD is about 1.9 times lower, indicating higher confidence in its average performance. More importantly, while achieving accuracy comparable to black-box models, LE-BRB retains the complete interpretability of BRB, and its diagnostic conclusions can be traced back to specific rules and their contributions, which is an advantage that black-box models do not possess.

#### 4.2.4. Parameter Sensitivity Analysis

**Figure 6:**
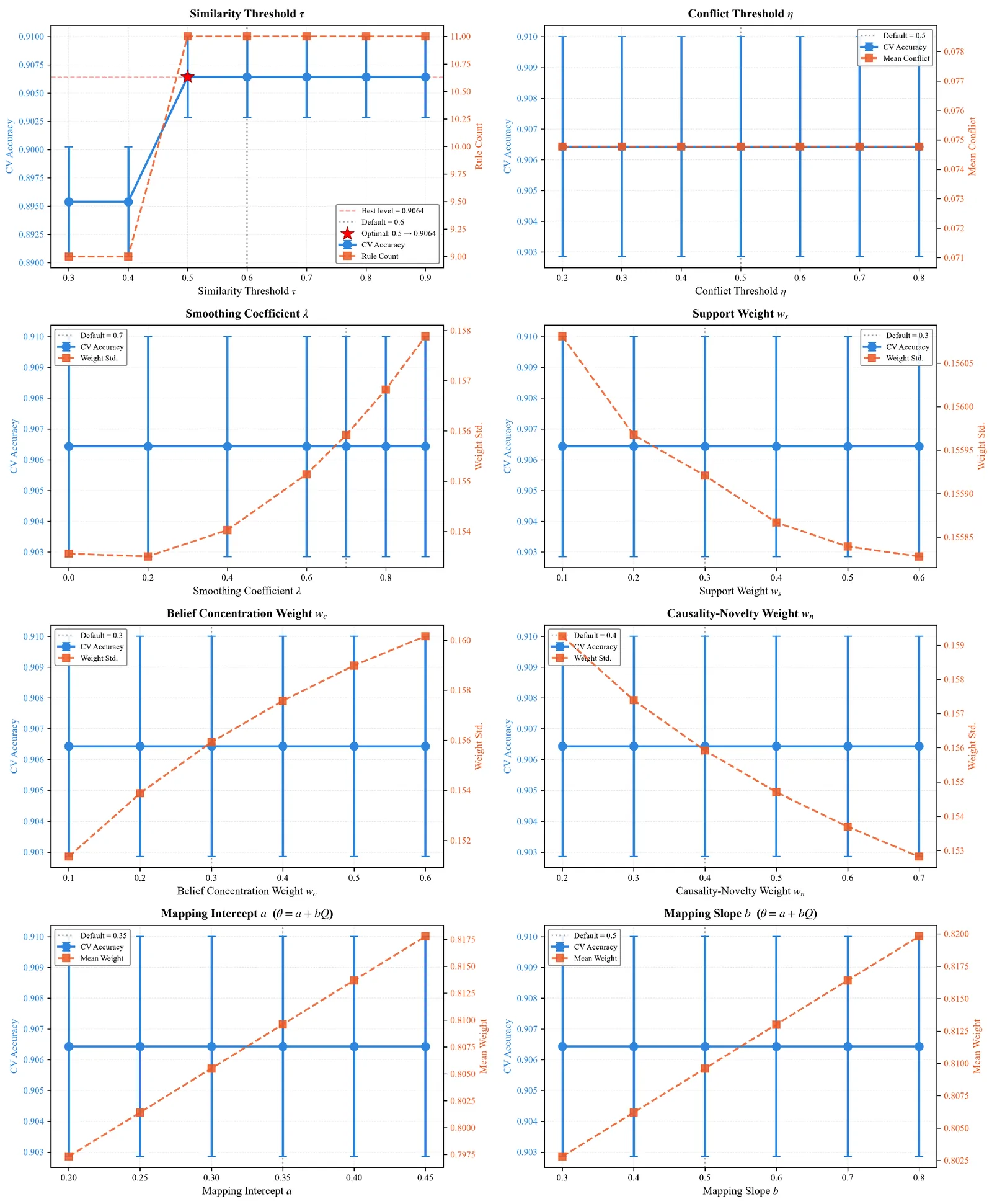
Parameter sensitivity analysis of LE-BRB on CWRU.

To verify the robustness of LE-BRB to key parameters, a univariate sensitivity analysis is conducted on eight parameters, including the similarity threshold τ, conflict threshold η, smoothing coefficient λ, support weight $w_{s}$, belief concentration weight $w_{c}$, causality-novelty weight $w_{n}$, mapping intercept *a*, and mapping slope *b*. Figure 6 shows the effect of each parameter variation on cross-validation accuracy and secondary metrics.

The results show that the cross-validation accuracy remains stable at approximately 0.9064 across most parameter variations, which falls within the range of the 10-round repeated random subsampling average accuracy of 0.9102±0.0051. The similarity threshold τ is the only parameter that significantly affects accuracy: when τ=0.3–0.4, the number of rules is about 9 and the accuracy is about 0.895; when τ≥0.5, the number of rules increases to 11 and the accuracy improves to 0.9064. Therefore, τ=0.5 is selected as the optimal value. The other parameters do not change the accuracy within the given ranges and mainly affect the rule weight distribution and the number of rules. Considering both accuracy and knowledge base consistency, the final parameter settings are as follows: τ=0.5, η=0.5, λ=0.7, $w_{s}=0.35$, $w_{c}=0.35$, $w_{n}=0.3$, a=0.35, and b=0.5.

### 4.3. MAFAULDA Bearing Dataset Experiment

#### 4.3.1. Ablation Study and Comparison with EBRB on the MAFAULDA First Dataset

To verify the performance of the proposed method in discovering missing faults in the knowledge base and generalizing across operating conditions, this section conducts validation on the MAFAULDA first and second datasets.

**Figure 7:**
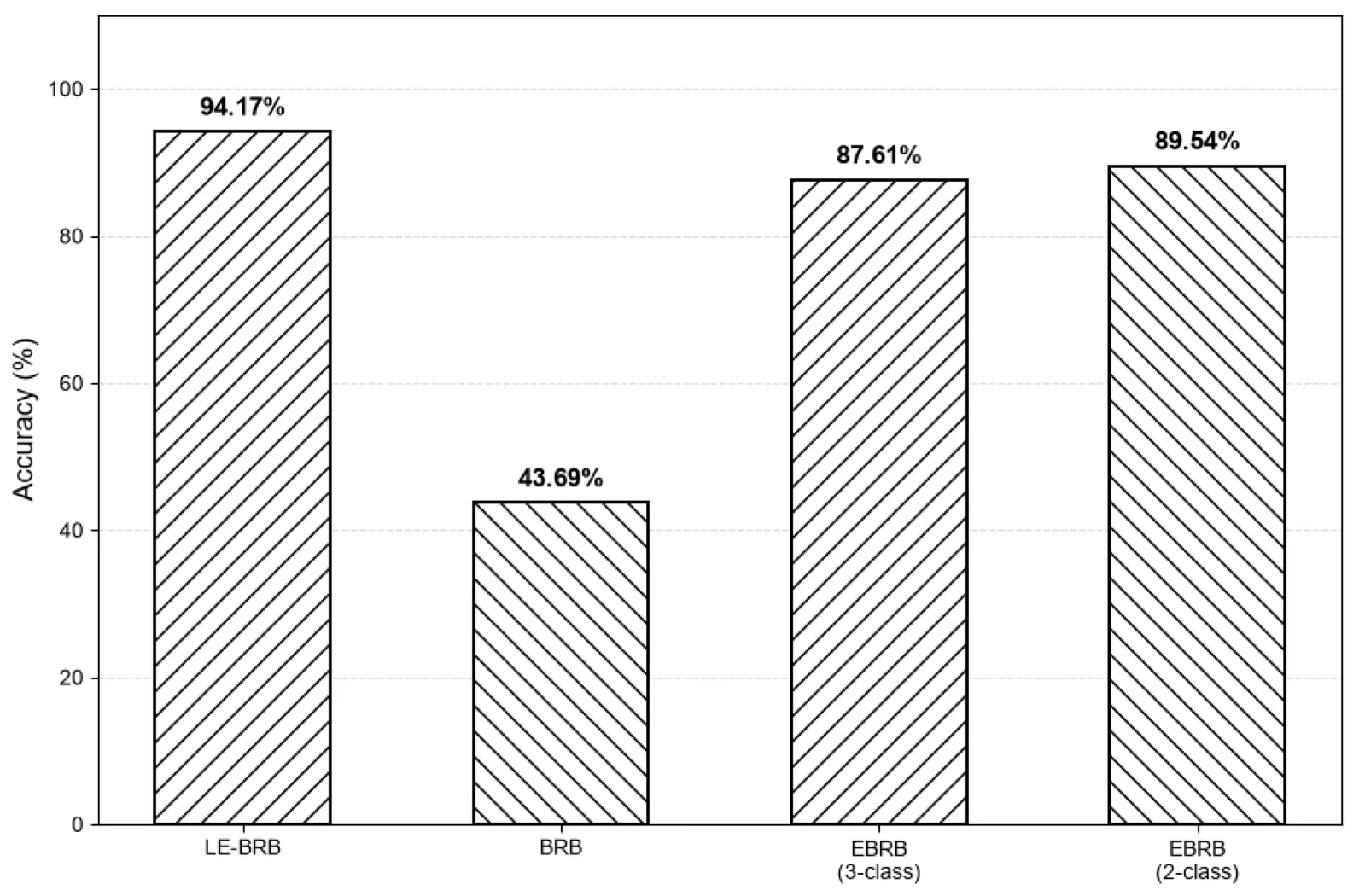
Comparison of LE-BRB, BRB, and EBRB on the MAFAULDA first dataset.

As shown in Figure 7 and Table 10, because the BRB cannot identify classes not modeled by the expert, its precision, recall, and F1 score on the unbalanced class are all 0, and other faults are misclassified as a result of inference failure. LE-BRB improves the overall accuracy from 43.69% to 94.17%. To further evaluate the advantage of autonomous rule expansion, two EBRB baselines are introduced: EBRB (3-class) with a closed set of three classes, and EBRB (2-class) with rejection. EBRB (3-class) pre-defines the unbalance class and achieves an overall accuracy of 87.61% with an F1-score of 0.92 on unbalance. For EBRB (2-class), the imbalance class is treated as an unknown category and rejected; the rejection rate for imbalance is 100%, and the known-class false rejection rate is 4.89%. In contrast, LE-BRB does not require pre-defining the missing class and still achieves the highest overall accuracy of 94.17% and an F1-score of 0.99 on unbalance. The conflict resolution strategy preserves the original expert knowledge, demonstrating that LE-BRB incorporates autonomous local updates of the knowledge base while retaining the excellent interpretability of BRB.

#### 4.3.2. Experimental Comparison on the MAFAULDA Dataset

**Figure 8:**
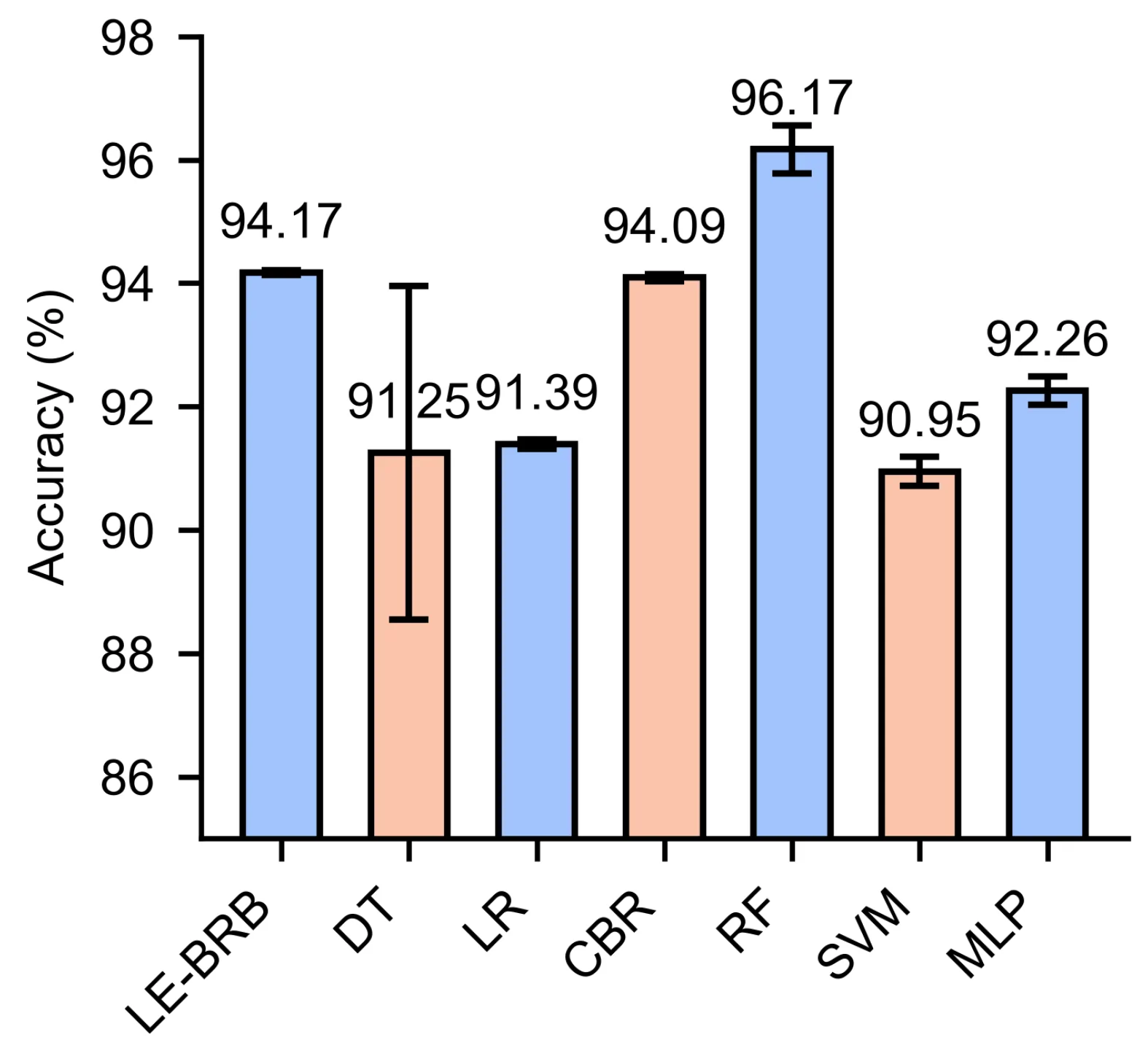
Accuracy comparison on the MAFAULDA first dataset.

As shown in Figure 8, LE-BRB achieves an accuracy of 94.17%, which is slightly higher than that of the white-box models DT, LR and CBR; it is lower than that of the black-box RF, but outperforms MLP and SVM, confirming that the model’s quality screening and conflict resolution can effectively capture the information gain from data rules. In terms of standard error of the mean (SEM), LE-BRB records only 0.000411, the lowest among all models and far below DT at 0.0271 and SVM at 0.0023. This indicates that LE-BRB attains optimal statistical stability through prior rule constraints while maintaining high accuracy.

As shown in Table 11 for the fault missing from the expert rule base, LE-BRB and all comparison models achieved an F1-score of 0.99 on the unbalance class, with recall and F1-score also reaching 99%, matching RF, MLP, DT and CBR, and outperforming SVM and LR, demonstrating a strong capability in identifying and detecting rule-missing categories; the normal class and the vertical misalignment known fault retained the original expert knowledge. It should be noted that although the imbalanced class reaches an F1-score of 0.99, the overall accuracy remains at 94.17% because the normal class, which accounts for 16.4% of the test set, attains a recall of only 0.69; the lower recall of the normal class, therefore, pulls down the class-weighted average accuracy. For the white-box models LR and DT, the recall for the normal class was 0.75, while the black-box RF achieved the best value of 0.87. Although LE-BRB underperformed RF in fine-grained metrics, its misclassification patterns are transparent and traceable, facilitating subsequent rule revision, an advantage that black-box models do not possess.

As shown in Table 12. Unlike the SEM, which measures the precision of the mean estimate, the SD and CV reflect the absolute and relative fluctuations of the model in a single output. The LE-BRB achieves the lowest values, with an SD of 0.0013 and a CV of 0.0014. The output of a purely data-driven model is a function of the training set partition; different partitions cause the model to converge to different local optima. By contrast, the output of the LE-BRB is determined by expert rules and is, therefore, insensitive to random partition fluctuations.

To verify the generalization of LE-BRB under the interpretability framework, data from the same dataset but with different specifications were selected, the semantic discretization partition was reset, and the rule structure itself remained unchanged.

**Figure 9:**
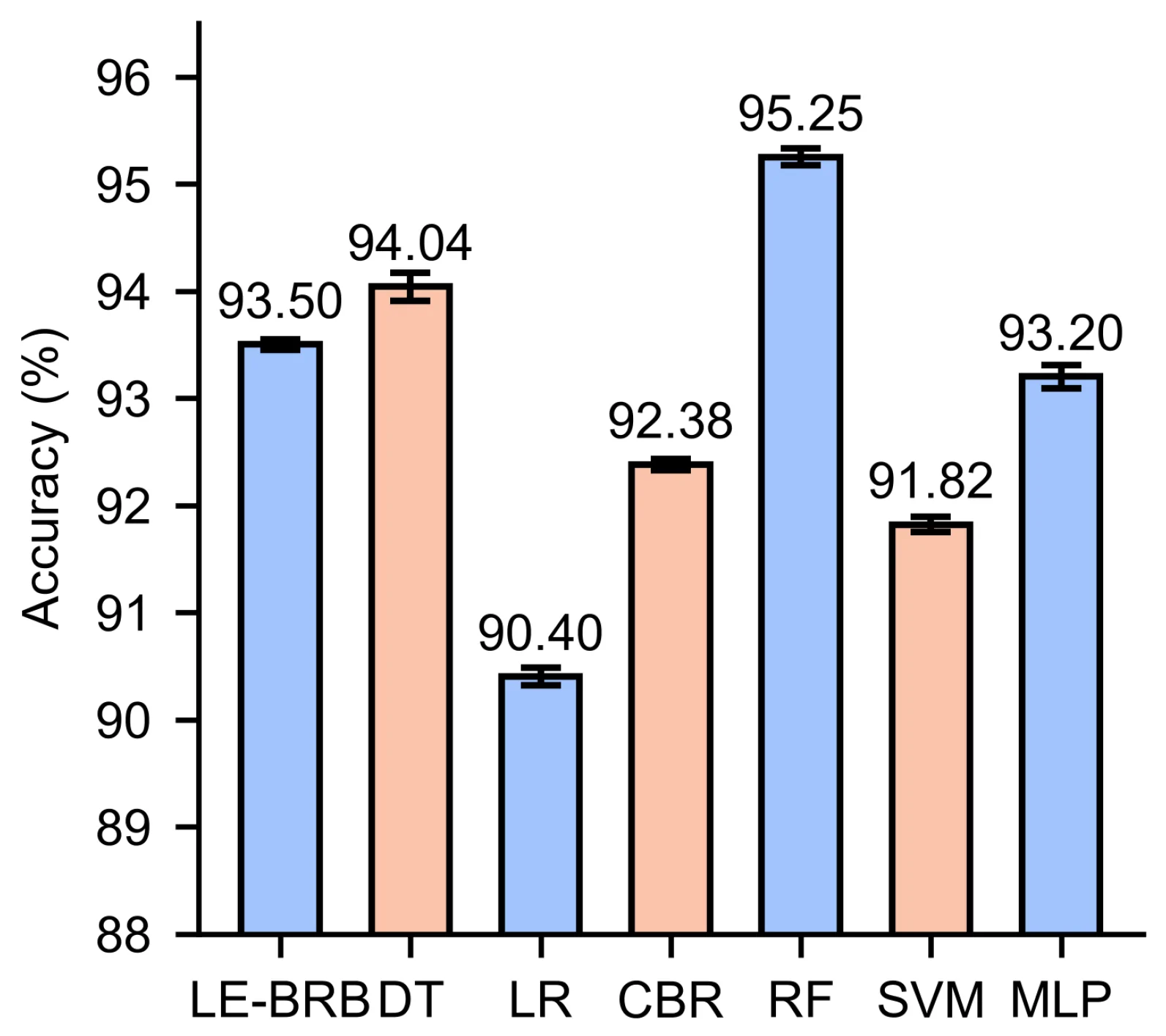
Accuracy comparison on the MAFAULDA second dataset.

As shown in Figure 9, in terms of overall accuracy, RF achieves the highest value, followed by DT and LE-BRB, while LR performs the weakest. However, the statistical confidence revealed by SEM presents a different pattern: the SEM of LE-BRB is only 0.000506, the lowest among all models, and is slightly better than those of DT and MLP, indicating that its mean estimation precision is extremely high. Although RF leads in accuracy, its SEM is approximately 1.6 times that of LE-BRB, suggesting that LE-BRB offers higher confidence when reporting average performance. This advantage originates from the belief rule base incorporating expert prior knowledge to constrain the parameter space, which effectively suppresses the mean shift caused by random sampling.

As shown in Table 13, for the unbalance class, all models except CBR and RF achieve an F1 score of 0.96 or above. For the vertical misalignment class, RF performs best, with a precision of 0.93, a recall of 0.96, and an F1 score of 0.94, followed closely by LE-BRB and MLP. For the normal class, RF achieves the highest F1 score of 0.93, followed by DT at 0.91 and LE-BRB at 0.90, which demonstrates the performance of the models under cross-condition conditions.

As shown in Table 14, LE-BRB achieves the lowest SD of 0.0016 and CV of 0.0017 among all models, outperforming the white-box models CBR and LR in terms of SD and CV, and remaining far lower than DT. Among the black-box models, the absolute and relative dispersion levels of RF, SVM, and MLP are all higher than those of LE-BRB. The extremely low SD and CV confirm that LE-BRB produces highly consistent outputs across repeated experiments, demonstrating excellent prediction consistency that is of considerable value for engineering reproducibility.

## 5. Conclusions

This paper addresses the problem that the conventional BRB cannot cover fault categories that exist in the training data but are not defined by experts, and proposes a learning-enhanced belief rule base (LE-BRB) diagnostic model. The main contributions and conclusions are as follows:

It breaks through the limitation that BRB relies on expert-predefined rule categories, automatically mining and learning categories not modeled by experts from data, thereby filling the gap in prior knowledge.

A complete process of low-support mining, multi-indicator validation, and conflict resolution is constructed to ensure the consistency and interpretability of the knowledge base.

On the CWRU local bearing fault dataset, LE-BRB successfully learns the rolling element fault (Ball) rules, achieving a 10-round cross-validation average accuracy of 91.02%, with a Ball-class F1-score of 0.65. On the MAFAULDA bearing dataset, LE-BRB successfully learns the rotor unbalance fault, improving the overall accuracy by more than 50 percentage points compared with the traditional BRB, and achieving 99% accuracy, recall, and F1-score for expert-unmodeled categories. While preserving the interpretability of the original BRB, LE-BRB achieves accuracy comparable to black-box models such as MLP, significantly outperforms white-box models such as decision trees, and exhibits relatively higher statistical stability than the compared models.

Although LE-BRB has been validated on CWRU and MAFAULDA for autonomous missing-rule supplementation and interpretable diagnosis, its current implementation is constrained by the exponential growth of the rule base with antecedent attributes. To control rule size and preserve transparent reasoning, only spectral centroid and kurtosis with expert-defined hard discretization are used; this trade-off helps suppress combinatorial explosion but may lose high-dimensional discriminative information under complex conditions and cause boundary effects near semantic boundaries. In addition, Pareto filtering has not been evaluated for 10+ dimensions, and the conflict-resolution mechanism prioritizes expert rules when premises are identical but conclusions conflict, without considering that expert rules may become outdated or biased due to equipment degradation or operating-condition drift, nor does it allow data-driven rules to override expert rules. Future work will introduce soft discretization, continuous association rule mining and rule reduction, investigate scalable/approximate Pareto filtering, and establish a dynamic expert-knowledge updating mechanism with trigger conditions and safety constraints for data-driven rules to override or update expert rules.

## Figures and Tables

**Table 1:** Dataset class distribution and class proportions.

Dataset	Class	Total	Training	Testing	Proportion
CWRU	Normal	471	141	330	40.0%
CWRU	Inner	236	71	165	20.1%
CWRU	Outer	236	71	165	20.1%
CWRU	Ball (missing)	234	70	164	19.9%
MAFAULDA 1	unbalance (missing)	5733	1720	4013	50.4%
MAFAULDA 1	misalignment	3773	1132	2641	33.2%
MAFAULDA 1	normal	1862	559	1303	16.4%
MAFAULDA 2	misalignment	4949	1485	3464	41.4%
MAFAULDA 2	unbalance (missing)	4606	1382	3224	38.5%
MAFAULDA 2	normal	2401	720	1681	20.1%

**Table 2:** Semantic discretization mapping of CWRU features.

Feature	Semantic Level	Symbol	Interval	Physical Meaning
Spectral centroid	Low	S1	<1157.83	Energy concentrated in low frequency
Spectral centroid	Medium-low	S2	1157.83–1364.68	Energy distributed in medium-low frequency
Spectral centroid	Medium-high	S3	1364.68–1385.43	Energy distributed in medium-high frequency
Spectral centroid	High	S4	>1385.43	Energy concentrated in high frequency
Kurtosis	Low	K1	<3.31	Smooth waveform, weak impulse
Kurtosis	Medium-low	K2	3.31–4.32	Slight impulse component
Kurtosis	Medium	K3	4.32–5.84	Moderate impulse component
Kurtosis	Medium-high	K4	5.84–6.06	Strong impulse component
Kurtosis	High	K5	>6.06	Strong impulse, typical fault feature

**Table 3:** Rule-level statistics.

Statistical Item	Value
Number of candidate rules	5
Number of Pareto front passes	3
Number finally accepted	3
Number eliminated	2
Number of conflict adjustments in generation stage	3
Whether expert rules remain unchanged	Yes
Expert rule weight	0.9
Range of learned rule weights	0.509–0.597

**Table 4:** Examples of mined Ball rules on CWRU.

Rule ID	Premise	Conclusion	Weight
learned_Ball_0	S4, K1	Ball (0.778), Normal (0.111), Inner (0.111)	0.597
learned_Ball_1	S3, K1	Ball (0.608), Normal (0.392)	0.568
learned_Ball_2	S2, K1	Ball (0.592), Normal (0.204), Outer (0.204)	0.509

**Table 5:** Comparison of LE-BRB and EBRB on CWRU (Ball and Inner classes).

Model	Ball			Inner		
	Precision	Recall	F1-Score	Precision	Recall	F1-Score
LE-BRB	0.94	0.49	0.65	1.00	1.00	1.00
BRB	0.00	0.00	0.00	1.00	1.00	1.00
EBRB (4-class)	0.81	0.65	0.72	1.00	1.00	1.00
EBRB (3-class)	0.01 ^1^	0.00 ^1^	0.00 ^1^	1.00	1.00	1.00

^1^ The Ball class is treated as unknown/rejected in the three-class open-set setting.

**Table 6:** Comparison of LE-BRB and EBRB on CWRU (Normal and Outer classes).

Model	Normal			Outer		
	Precision	Recall	F1-Score	Precision	Recall	F1-Score
LE-BRB	0.80	0.98	0.88	1.00	1.00	1.00
BRB	0.67	1.00	0.80	1.00	1.00	1.00
EBRB (4-class)	0.84	0.94	0.89	1.00	0.95	0.98
EBRB (3-class)	0.67	1.00	0.80	1.00	0.78	0.88

**Table 7:** Comparison of metrics on CWRU (Ball and Inner classes).

Model	Ball			Inner		
	Precision	Recall	F1-Score	Precision	Recall	F1-Score
LE-BRB	0.94	0.49	0.65	1.00	1.00	1.00
RF	0.80	0.73	0.76	1.00	1.00	1.00
SVM	0.84	0.72	0.77	1.00	1.00	1.00
MLP	0.34	0.18	0.20	1.00	0.95	0.97
DT	0.74	0.79	0.76	1.00	1.00	1.00
LR	0.84	0.68	0.75	1.00	1.00	1.00
CBR	0.79	0.74	0.76	1.00	1.00	1.00

**Table 8:** Comparison of metrics on CWRU (Normal and Outer classes).

Model	Normal			Outer		
	Precision	Recall	F1-Score	Precision	Recall	F1-Score
LE-BRB	0.84	0.94	0.89	1.00	0.95	0.98
RF	0.87	0.91	0.89	1.00	1.00	1.00
SVM	0.87	0.93	0.90	1.00	1.00	1.00
MLP	0.71	0.98	0.82	1.00	1.00	1.00
DT	0.89	0.85	0.87	0.99	1.00	1.00
LR	0.85	0.94	0.89	1.00	1.00	1.00
CBR	0.87	0.90	0.89	1.00	1.00	1.00

**Table 9:** Comparison of stability on CWRU.

Model	LE-BRB	DT	LR	CBR	RF	SVM	MLP
SD	0.0051	0.0164	0.0041	0.0063	0.0090	0.0056	0.0497
CV	0.0056	0.0182	0.0045	0.0069	0.0099	0.0061	0.0608

**Table 10:** Comparison of metrics between LE-BRB, BRB, and EBRB on the MAFAULDA first dataset.

Model	Unbalance			Vertical Misalignment			Normal		
	Precision	Recall	F1-Score	Precision	Recall	F1-Score	Precision	Recall	F1-Score
LE-BRB	0.99	0.99	0.99	0.86	0.98	0.98	0.94	0.69	0.79
BRB	0.00	0.00	0.00	0.86	0.98	0.90	0.18	0.69	0.29
EBRB (3-class)	1.00	0.84	0.92	0.73	0.98	0.84	0.86	0.68	0.76
EBRB (2-class)	—	—	—	0.85	0.96	0.90	0.98	0.58	0.73

For EBRB (2-class), the unbalance class is treated as unknown and rejected; its F1-score is 0.98 for the Unknown class, and the known-class false rejection rate is 4.89%. The symbol “—” indicates that the corresponding class is not predicted as a specific fault category.

**Table 11:** Comparison of metrics on the MAFAULDA first dataset.

Model	Unbalance			Vertical Misalignment			Normal		
	Precision	Recall	F1-Score	Precision	Recall	F1-Score	Precision	Recall	F1-Score
LE-BRB	0.99	0.99	0.99	0.86	0.98	0.98	0.94	0.69	0.79
RF	0.99	0.99	0.99	0.91	0.98	0.98	0.96	0.80	0.87
SVM	0.99	0.96	0.98	0.82	0.98	0.98	0.94	0.69	0.79
MLP	0.99	0.99	0.99	0.87	0.98	0.98	0.95	0.71	0.81
DT	0.99	0.99	0.99	0.88	0.93	0.93	0.84	0.75	0.79
LR	0.99	0.96	0.98	0.85	0.94	0.94	0.83	0.75	0.79
CBR	0.99	0.99	0.99	0.89	0.94	0.94	0.86	0.78	0.83

**Table 12:** Comparison of stability on the MAFAULDA first dataset.

Model	LE-BRB	DT	LR	CBR	RF	SVM	MLP
SD	0.0013	0.0857	0.0023	0.0017	0.0123	0.0073	0.0072
CV	0.0014	0.0939	0.0025	0.0018	0.0133	0.0081	0.0075

**Table 13:** Comparison of metrics on the MAFAULDA second dataset.

Model	Unbalance			Vertical Misalignment			Normal		
	Precision	Recall	F1-Score	Precision	Recall	F1-Score	Precision	Recall	F1-Score
LE-BRB	0.99	0.92	0.96	0.90	0.97	0.93	0.89	0.90	0.90
DT	0.99	0.95	0.97	0.90	0.96	0.93	0.95	0.88	0.91
LR	0.99	0.86	0.92	0.82	0.99	0.90	0.97	0.80	0.87
CBR	0.97	0.93	0.95	0.87	0.97	0.92	0.96	0.82	0.89
RF	0.94	0.96	0.95	0.93	0.96	0.94	0.95	0.91	0.93
SVM	0.99	0.89	0.94	0.84	0.99	0.91	0.99	0.81	0.89
MLP	0.99	0.93	0.96	0.91	0.96	0.93	0.99	0.81	0.89

**Table 14:** Comparison of stability on the MAFAULDA second dataset.

Model	LE-BRB	DT	LR	CBR	RF	SVM	MLP
SD	0.0016	0.0042	0.0026	0.0017	0.0025	0.0023	0.0035
CV	0.0017	0.0047	0.0029	0.0018	0.0026	0.0025	0.0038

## Data Availability

The CWRU bearing fault dataset used in this study is publicly available from the Case Western Reserve University Bearing Data Center https://engineering.case.edu/bearingdatacenter (accessed on 15 June 2026). A comprehensive benchmark study of this dataset is provided by [27]. The MAFAULDA bearing fault dataset used in this study is publicly available online (no DOI assigned) from the Signals, Multimedia and Telecommunications Laboratory (SMT) of the Federal University of Rio de Janeiro (UFRJ), Brazil. The dataset can be downloaded at: https://www02.smt.ufrj.br/~offshore/mfs/ (accessed on 15 June 2026).
